# Promoter Hypomethylation of TGFBR3 as a Risk Factor of Alzheimer’s Disease: An Integrated Epigenomic-Transcriptomic Analysis

**DOI:** 10.3389/fcell.2021.825729

**Published:** 2022-03-02

**Authors:** Hui Song, Jue Yang, Wenfeng Yu

**Affiliations:** ^1^ Key Laboratory of Endemic and Ethnic Diseases, Ministry of Education and Key Laboratory of Medical Molecular Biology of Guizhou Province, School of Basic Medical Sciences, Guizhou Medical University, Guiyang, China; ^2^ The State Key Laboratory of Functions and Applications of Medicinal Plants, Guizhou Medical University, Guiyang, China; ^3^ The Key Laboratory of Chemistry for Natural Products of Guizhou Province and Chinese Academic of Sciences, Guiyang, China

**Keywords:** Alzheimer’s disease, methylation, TGFBR3, Aβ plaque, secretase activity

## Abstract

Alzheimer’s disease (AD) is characterized by the abnormal deposition of amyloid-β (Aβ) plaques and tau tangles in the brain and accompanied with cognitive impairment. However, the fundamental cause of this disease remains elusive. To elucidate the molecular processes related to AD, we carried out an integrated analysis utilizing gene expression microarrays (GSE36980 and GSE5281) and DNA methylation microarray (GSE66351) in temporal cortex of AD patients from the Gene Expression Omnibus (GEO) database. We totally discovered 409 aberrantly methylated and differentially expressed genes. These dysregulated genes were significantly enriched in biological processes including cell part morphogenesis, chemical synaptic transmission and regulation of Aβ formation. Through convergent functional genomic (CFG) analysis, expression cross-validation and clinicopathological correlation analysis, higher TGFBR3 level was observed in AD and positively correlated with Aβ accumulation. Meanwhile, the promoter methylation level of TGFBR3 was reduced in AD and negatively associated with Aβ level and advanced Braak stage. Mechanically, TGFBR3 might promote Aβ production by enhancing β- and γ-secretase activities. Further investigation revealed that TGFBR3 may exert its functions via Synaptic vesicle cycle, Calcium signaling pathway and MAPK signal pathway by regulating hub genes GNB1, GNG3, CDC5L, DYNC1H1 and FBXW7. Overall, our findings highlighted TGFBR3 as an AD risk gene and might be used as a diagnostic biomarker and therapeutic target for AD treatment.

## Introduction

Alzheimer’s disease (AD) ranks as the leading cause of dementia, with an estimated 60–80% of cases worldwide (Alzheimer’s Association, 2021). The neuropathologic hallmarks of AD are the deposition of extracellular amyloid-β (Aβ) plaques and intracellular tau-containing neurofibrillary tangles (Busche and Hyman, 2020). Although multiple lines of evidence clearly point to Aβ as a critical disease initiator, most clinical trials of anti-Aβ therapies have failed to substantially improve clinical symptoms (Mangialasche et al., 2010; Cummings et al., 2019; Lozupone et al., 2020), highlighting the need for a better understanding of the AD etiology.

AD is influenced by both genetic and environmental factors. Large-scale genome-wide association studies have successfully identified many AD-associated genetic variants, such as ATP8B1 rs2571244, DLGAP2 at chr8: 1316870 and ADAM17 rs142946965 (Dumitrescu et al., 2020; Hartl et al., 2020; Ouellette et al., 2020). However, the common variants illustrate only 3–4% genetic heritability for each locus and much of the heritability of AD could not been fully explained by measured loci (Lord and Cruchaga, 2014). Therefore, the pathogenic role of nongenetic factors in AD, especially sporadic AD, has attracted extensive attention. About one third of AD patients are affected by a variety of nongenetic factors, most of which are related to environment and lifestyle, such as radiation, bacterial infection, education, stress, diet, smoking and drinking (Ngandu et al., 2015; Dunn et al., 2019; Sierksma et al., 2020). However, the molecular mechanism of how these environmental factors affect the AD occurrence has not yet been clarified.

Epigenetics has been regarded as the genetic response to the environmental agents and lifestyle factors, in the way that modify gene expression without any changes in DNA sequence (Xiao et al., 2020). DNA methylation, one of the most common well-described epigenetic modifications, has been tightly linked to transcript expression changes (Greenberg and Bourc’his, 2019). Hypermethylation of CpG islands in promoter regions is usually associated with transcriptional silencing. Previous studies have demonstrated that abnormally methylated genes had major roles during AD neuropathology. For instance, PSEN1 gene encodes the catalytic peptide of the γ-secretase complex that regulates Aβ processing and accumulation (Hass et al., 2009). Both CpG and non-CpG hypomethylation of PSEN1 promoter was reported to be significantly associated with PSEN1 expression in AD (Monti et al., 2020). In addition, Sanchez-Mut et al. observed that promoter hypermethylation caused a reduced expression of DUSP22 in the hippocampus of AD patients, and DUSP22 depletion could inhibit tau Thr231 phosphorylation and activated CREB signal by increasing the phosphorylation of PKA thr197 (Sanchez-Mut et al., 2014). Furthermore, dysregulated promoter methylation also has a role in neuron development as well as alternative splicing and promoter usage relevant to AD pathogenesis (Mills et al., 2013; Torok et al., 2017; Caldwell et al., 2020). Despite that, the methylation-affected genes and their functional roles during AD remain largely unclear. Herein, more studies are needed to comprehensively understand the methylation profile and mechanisms underlying their associations with AD pathobiology.

Gene Expression Omnibus (GEO) is a public functional genomics data repository that archives microarray and high throughput sequencing data (Barrett et al., 2013). To date, the database hosts more than 158,000 public series and comprises 4,560,000 samples covering various human diseases, including AD. In this work, we first analyzed the DNA methylation profiles and gene expression levels in temporal cortex from patients with AD and normal controls using GEO datasets. Functional enrichment analysis was performed for the aberrantly methylated and differentially expressed genes. Convergent functional genomic (CFG) analysis was used to identified candidate genes involved in AD pathogenesis. Expression level of candidate genes in different brain regions was then validated. Among them, we identified TGFBR3 expression was upregulated in AD while its promoter methylation level of cg17074213 was significantly downregulated. Furthermore, we investigated the association between TGFBR3 and AD pathocharacteristic features, and also explored the possible mechanisms.

## Materials and Methods

### Microarray Data Profile

The DNA methylation dataset GSE66351 (methylation dataset 1) and gene expression datasets GSE36980 (expression dataset 1) and GSE5281 (expression dataset 2) were collected from the GEO. Methylation dataset 1 contains 39 AD temporal cortex, 26 normal temporal cortex based on the platform GPL13534 (Illumina HumanMethylation450 BeadChip) (Gasparoni et al., 2018). Expression dataset 1 has 10 AD temporal cortex and 19 non-AD temporal cortex based on the platform GPL6244 (Affymetrix Human Gene 1.0 S T Array) while expression dataset 2 has 16 AD temporal cortex and 12 normal temporal cortex based on the platform GPL570 (Affymetrix Human Genome U133 Plus 2.0 Array) (Liang et al., 2007; Hokama et al., 2014).

### Data Processing

GEO2R, an interactive web tool to perform comparisons on the microarray data, was utilized to identify differentially methylated genes and differentially expressed genes. For both differentially methylated genes and differentially expressed genes, *p* < 0.05 and |t| > 2 were used as the cutoff criteria. Hypomethylated-upregulated genes were obtained by overlapping hypomethylated and highly expressed genes, while hypermethylated-downregulated genes were obtained by overlapping hypermethylated and lowly expressed genes using the software FunRich 3.1.3 (Fonseka et al., 2021).

### Gene Functional and Pathway Enrichment Analysis

Gene Ontology (GO) for biological process and Kyoto Encyclopedia of Genes and Genomes (KEGG) pathway enrichment analysis was performed using a gene annotation and analysis resource Metascape (Zhou et al., 2019). Terms with *p*-value less than 0.05 were considered to be statistically significant.

### Protein-Protein Interaction (PPI) Network Construction and Hub Genes Identification

PPI network was generated using STRING v11.5 (Szklarczyk et al., 2021) and visualized by Cytoscape v3.8.2 (Shannon et al., 2003). The interaction score was set as highest confidence 0.9. The MCODE was identified using Metascape from the network. Then the degree score of each gene in the network was calculated by cytoHubba (Chin et al., 2014). The 10 genes with the highest degree score were identified as hub genes.

### CFG Analysis

CFG approach was used to integrated evaluate the association of a gene with AD-related multiple evidence, including expressional quantitative trait loci (eQTL), genome-wide association study (GWAS), PPI, correlation with AD pathology Aβ and Tau, and early differentially expressed genes (Xu et al., 2018). If any of the features mentioned above were presented, one point of CFG score was assigned. The CFG score was ranged from 0 to 5 points. A higher CFG score of a gene means a stronger correlation with AD pathogenesis.

Gene expression validation, and its correlation with clinical features and secretase activities.

Expression profiles of candidate genes in four brain regions (entorhinal cortex, hippocampus, temporal cortex and frontal cortex) were obtained from cross platform normalized data in AlzData (Xu et al., 2018). The relationship between gene expression and Aβ aggregation as well as tau hyperphosphorylation were analyzed in 68 human brain temporal cortex tissues from the Allen Brain Atlas (http://www.brain-map.org) (Miller et al., 2017). The associations of TGFBR3 with Aβ42 level, Braak stage, α-, β- and γ-secretase activities, age, gender and APOE genotype were evaluated using GSE106241 dataset (expression dataset 3) which contains 55 AD temporal cortical samples (Marttinen et al., 2019). A *p*-value less than 0.05 were considered to be statistically significant. The TGFBR3-related genes were also obtained from the expression dataset 3 according to the cut-off standards |r|>0.6 and *p* < 0.05.

### Statistical Analysis

All statistical analysis and data visualizations were performed using GraphPad Prism 9.0. The differences between two continuous variables were analyzed by Student’s t-test. Pearman method was applied for correlation analysis. *p* < 0.05 was considered statistically significant.

## Results

### Identification of Aberrantly Methylated and Differentially Expressed Genes in Temporal Cortex of Patients With AD

**FIGURE 1:**
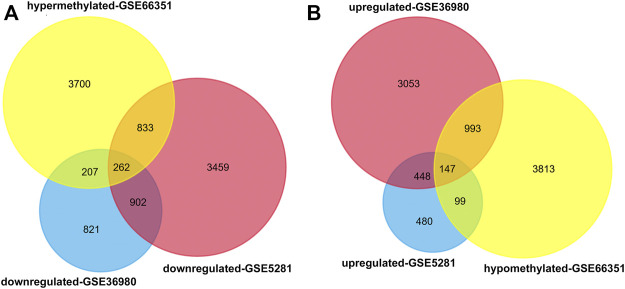
Identification of aberrantly methylated and differentially expressed genes in temporal cortex of patients with AD. GEO2R was used to analyze the DNA methylation profiling of methylation dataset 1 and gene expression profiling of expression dataset 1 and 2. The aberrantly methylated genes and differentially expressed genes were identified according to the cutoff criteria |t| > 2 and *p* < 0.05. Then Venn diagram analysis was performed to aberrantly methylated and differentially expressed genes in the three datasets **(A)** Hypermethylated-downregulated genes **(B)** Hypomethylated-upregulated genes.

Using the online software GEO2R, we obtained 10,054 aberrantly methylated genes from methylation dataset 1, including 5,002 hypermethylated and 5,052 hypomethylated. For expression profiles, we analyzed the expression dataset 1 and 2. After filtering analysis, 3,366 and 10,097 differentially expressed genes were retrieved from the two datasets, respectively. Venn diagram analysis identified 409 methylation affected genes, including 262 hypermethylated-downregulated genes and 147 hypomethylated-upregulated genes (Figures 1A,B).

### Gene Functional Enrichment Analysis

**FIGURE 2:**
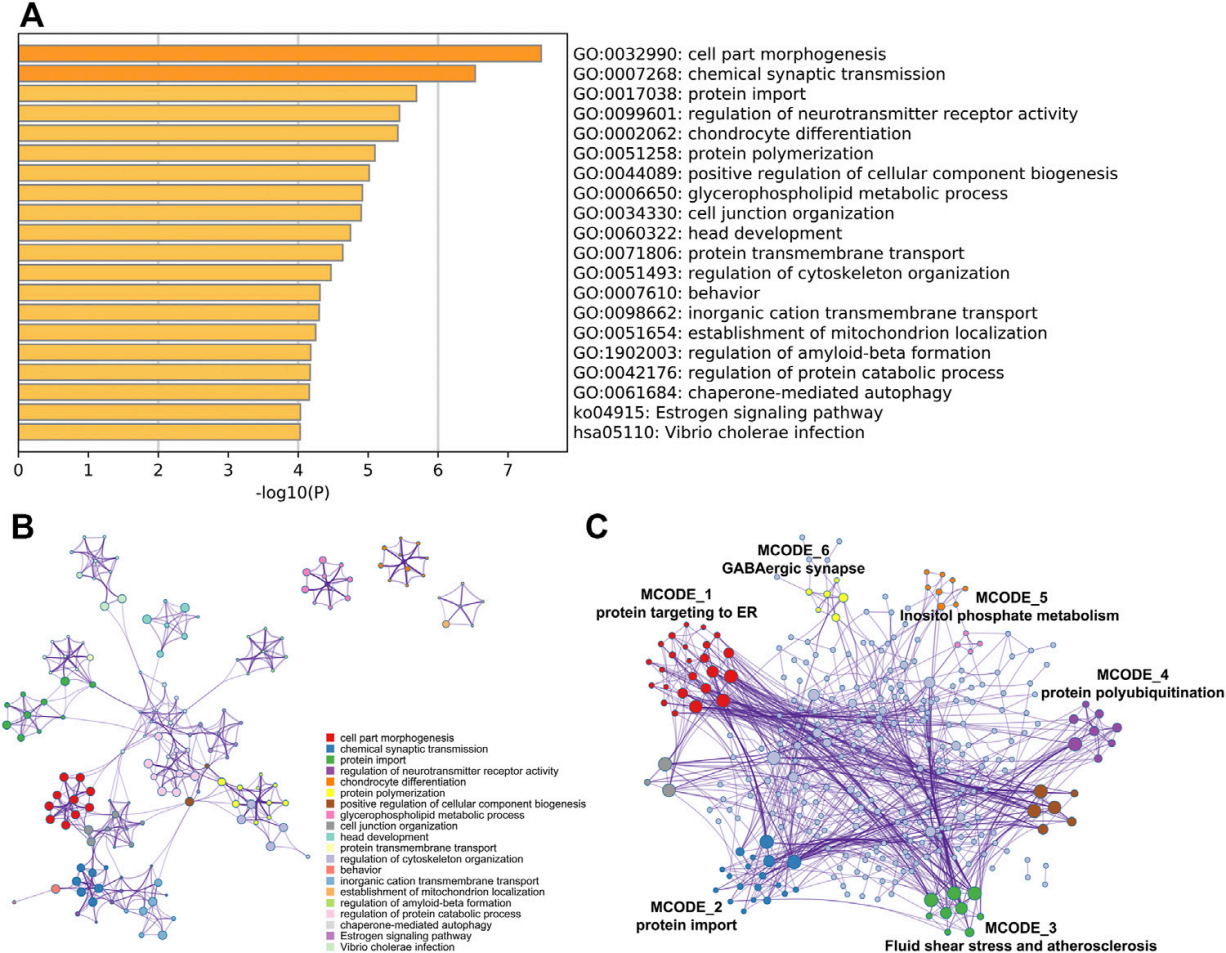
Gene functional enrichment analysis. GO annotation and KEGG pathway enrichment analysis of the aberrantly methylated and differentially expressed genes was analyzed by Metascape **(A)** Bar graph of the top 20 enriched terms across, colored by *p*-values **(B)** Network of the top 20 enriched terms and **(C)** MCODE components was identified from the PPI network.

To investigate the potential effects of abnormal methylation on the AD pathogenesis, GO annotation and KEGG pathway enrichment analysis of the 409 methylation affected genes were conducted by online tool Metascape. As showed in Figure 2A, cell part morphogenesis, chemical synaptic transmission, protein import, regulation of neurotransmitter receptor activity and chondrocyte differentiation were statistically enriched. Interestingly, the aberrantly methylated and differentially expressed genes was also obviously associated with regulation of Aβ formation (*p* = 6.63E-05), which has been considered as a key risk factor of AD initiation. Cluster network analysis of the enriched items indicated that most items were closely interrelated except glycerophospholipid metabolic process, chondrocyte differentiation and establishment of mitochondrion localization (Figure 2B). PPI network was then constructed and MCODE algorithm was applied to identify neighborhoods where proteins are densely connected. From the PPI network, six MCODE network were identified, including protein targeting to ER, protein import, Fluid shear stress and atherosclerosis, protein polyubiquitination, Inositol phosphate metabolism and GABAergic synapse (Figure 2C).

### Candidate Gene Identification and Expression Cross-Validation

**FIGURE 3:**
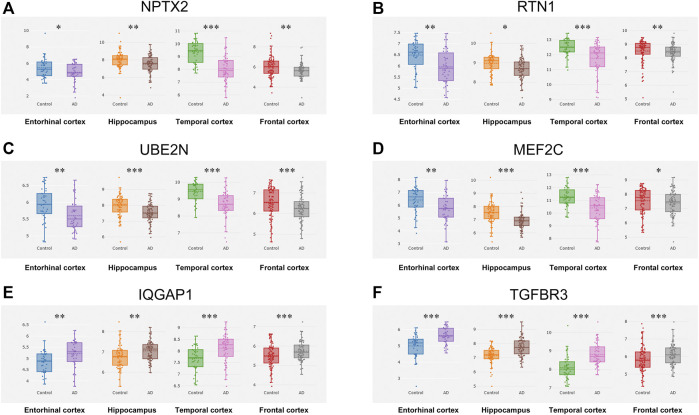
Expression cross-validation of the candidate genes in AlzData database **(A)** NPTX2 **(B)** RTN1 **(C)** UBE2N **(D)** MEF2C **(E)** IQGAP1 **(F)** TGFBR3. *p < 0.05, **p < 0.01, ***p < 0.001.

The CFG approach was used to score candidate genes based on their association with AD risk factors, including genetic association of DNA variations with disease susceptibility, gene expression regulated by AD genetic variants, protein interaction with AD core proteins, and diagnosis prediction of disease models. After prioritization for each gene, 17 genes showed a high level of AD relevance (CFG score = 4, Table 1). Importantly, expression levels of all 17 genes were differentially expressed in AD mouse models before AD pathology emergence, suggesting that these genes could be used as early indicators for AD diagnosis. Then, we validated their expression levels in the entorhinal cortex, hippocampus, temporal cortex and frontal cortex. Compared with control tissues, expression levels of NPTX2, RTN1, UBE2N and MEF2C were significantly decreased, while expression levels of IQGAP1 and TGFBR3 were increased in all the four different brain regions of patients with AD (Figure 3).

**TABLE 1:** CFG analysis of the candidate genes in AlzData database.

Gene	EQTL	GWAS	PPI	Early DEG	Pathology cor (aβ)	Pathology cor (tau)	CFG
MARK1	4	1	—	yes	−0.317,*	−0.709,**	4
NPTX2	1	1	—	yes	−0.688,***	−0.783,***	4
TRHR	3	0	APP, PSEN1	yes	−0.434,**	0.318,ns	4
EPHA4	3	4	MAPT	yes	−0.238,ns	0.190,ns	4
RTN1	2	2	—	yes	−0.704,***	−0.647,**	4
UBE2N	1	0	PSEN2	yes	−0.652,***	−0.832,***	4
MYT1L	3	12	—	yes	−0.488,***	−0.583,*	4
MEF2C	1	2	PSEN2, MAPT	yes	−0.159,ns	−0.198,ns	4
HSBP1	1	0	MAPT	yes	−0.409,**	0.083,ns	4
IFITM3	2	0	PSEN2, MAPT	yes	0.863,***	0.615,*	4
PLEKHA7	3	0	PSEN1	yes	−0.443,**	−0.672,**	4
IQGAP1	1	0	PSEN1	yes	0.310,*	0.282,ns	4
CLU	0	74	APP	yes	0.811,***	0.546,*	4
RHOG	1	0	PSEN2	yes	0.592,***	0.015,ns	4
IFNGR1	2	0	MAPT	yes	0.749,***	0.698,**	4
LRIG1	7	1	—	yes	0.500,***	0.253,ns	4
TGFBR3	1	25	—	yes	0.527,***	0.399,ns	4

eQTL, expressional quantitative trait loci; GWAS, genome-wide association study; DEG, differentially expressed gene; cor, correlation.

### High TGFBR3 Level Was Significantly Associated With Aβ Accumulation

**FIGURE 4:**
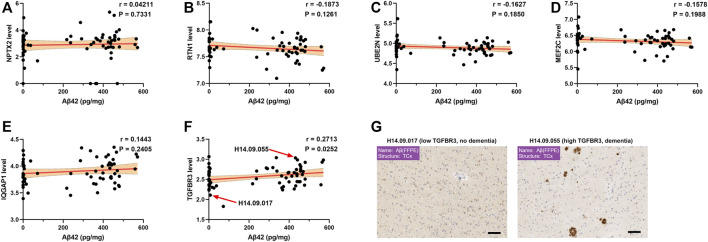
High TGFBR3 level was significantly associated with Aβ accumulation **(A-F)** Pearson correlation analysis was used to evaluate the association between Aβ level and gene expression levels of NPTX2 **(A)**, RTN1 **(B)**, UBE2N **(C)**, MEF2C **(D)**, IQGAP1 **(E)** and TGFBR3 **(F)** in 68 brain temporal cortex tissues from the Allen Brain Atlas **(G**) Representative immunohistochemistry staining data for Aβ. Scale bars, 100 μm.

Aβ aggregation and tau hyperphosphorylation are two major pathological features of AD. Thus, we investigated the relationship between gene expression and Aβ aggregation as well as tau hyperphosphorylation in the human brain data derived from the Allen Brain Atlas. Among the six candidate genes, there was a strong positive correlation of TGFBR3 with Aβ level (r = 0.2713, *p* = 0.0252, Figure 4F). However, Aβ level was not correlated with NPTX2, RTN1, UBE2N, MEF2C and IQGAP1 expression (Figures 4A–E). Immunohistochemistry staining of formalin fixation and paraffin embedding brain tissues showed that patients with a high TGFBR3 level manifested dementia and obvious Aβ deposition, but patients with low TGFBR3 level did not exhibit dementia and accumulate Aβ (Figure 4G). However, none of the six genes showed statistical correlation with phosphorylated tau level (Supplementary Figure S1).

### The Promoter Methylation Level of TGFBR3 was Reduced in AD and Negatively Correlated with Advanced Braak Stage

**FIGURE 5:**
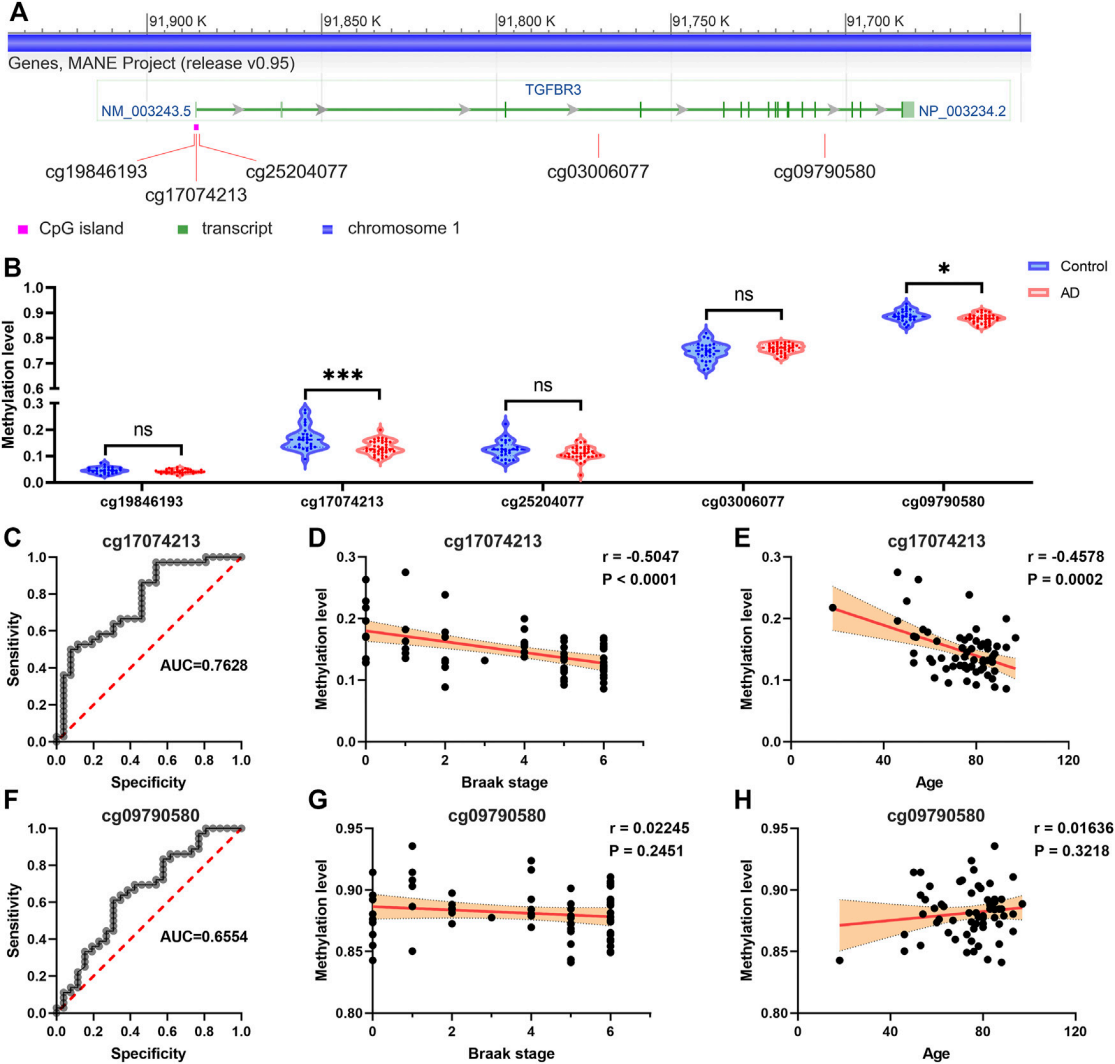
The promoter methylation level of TGFBR3 was reduced in AD and negatively correlated with advanced Braak stage **(A)** The methylation probes of TGFBR3 (NM_003,243.5) annotated by the USUC genome browser **(B)** Methylation levels of methylation probes for TGFBR3 in AD tissues and controls from methylation dataset 1 **(C)** The ROC curves for predicting AD by the cg17074213 **(D)** The correlation analysis of cg17074213 level and Braak stage **(E)** The correlation analysis of cg17074213 level and age **(F)** The ROC curves for predicting AD by the cg09790580 **(G)** The correlation analysis of cg09790580 level and Braak stage **(H)** The correlation analysis of cg09790580 level and age. ns: not significant, *p < 0.05, ***p < 0.001.

According to the USUC genome browser annotation, we discovered 5 methylation probes in the TGFBR3 (Figure 5A). Among them, cg17074213 is a promoter-associated methylation site in high-CpG island and located in the first exon or 5′UTR of TGFBR3. Compared with control tissues, The methylation levels of cg17074213 and cg09790580 in AD tissues were dramatically downregulated based on methylation dataset 1 (Figure 5B). The area under the curve (AUC) of cg17074213 and cg09790580 was 0.7628 and 0.6554, suggesting that cg17074213 had better potential diagnostic value in distinguishing AD and normal samples (Figures 5C,F). We also analyzed their correlations between the methylation level and pathological features of patients in methylation dataset 1 and found that cg17074213 was strikingly associated with Braak stage and age (Figures 5D,E), but less correlation was observed for cg09790580 (Figures 5G,H).

### TGFBR3 Expression Was Positively Correlated With β- and γ-secretase Activities

**FIGURE 6:**
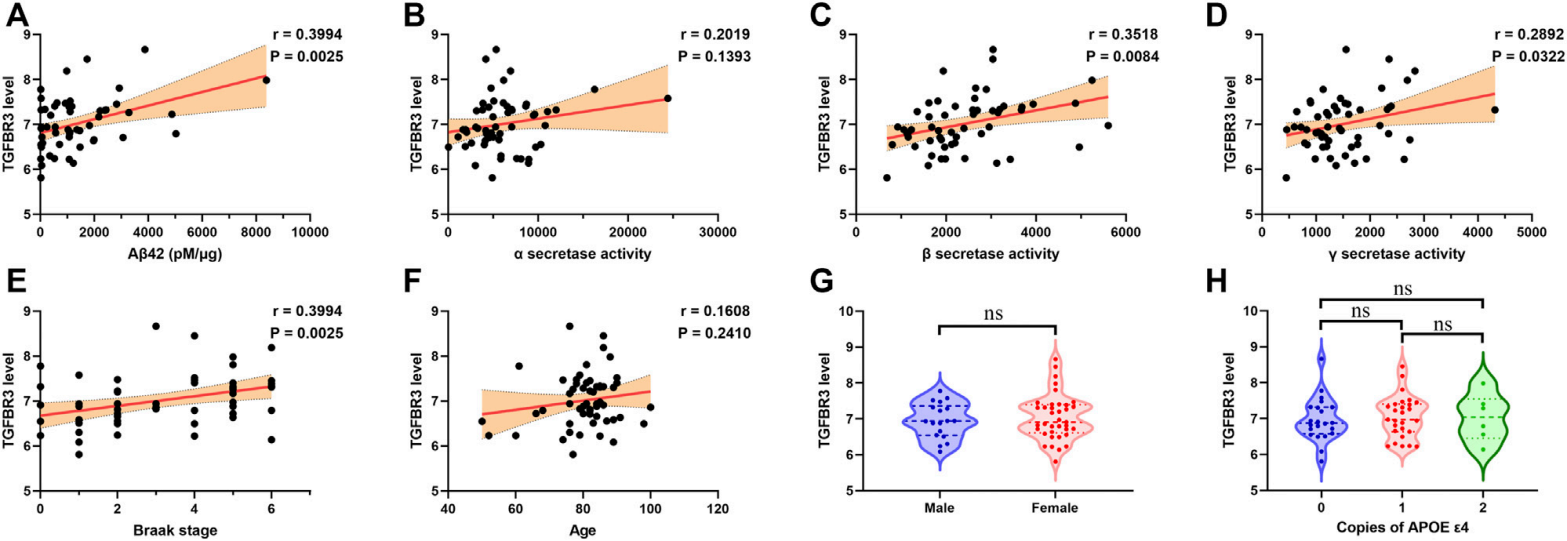
TGFBR3 expression positively correlated with β- and γ-secretase activities **(A-F)** Pearson correlation analysis was used to evaluate the association between TGFBR3 level and Aβ42 level **(A)**, α-secretase activity **(B)**, β-secretase activity **(C)**, γ-secretase activity **(D)**, Braak stage **(E)** and Age **(F)** in 55 brain temporal cortex tissues from the expression dataset 3 **(G)** TGFBR3 level in male and female **(H)** TGFBR3 level in different APOE genotypes. ns *p* > 0.05.

Analysis of the expression dataset 3 from the GEO database confirmed a positive correlation between TGFBR3 expression and Aβ42 level in temporal cortex of patients with AD (*p* = 0.0025; Figure 6A). The neurotoxic Aβ peptides are generated via serial cleavage of amyloid precursor protein (APP) by β-secretase and γ-secretase (Kent et al., 2020). On the contrary, α-secretase cleaves APP to yield sAPPα, a neuroprotective fragment. At this point, altered activity of these secretases could determine the form of APP cleavage and lead to different functional consequences. We then evaluated possible associations between the expression of TGFBR3 and different secretase activities. Correlation analysis indicated that high level of TGFBR3 was prominently related to β- and γ-secretase activities, but showed low association with α-secretase activities (Figures 6B–D). Furthermore, patients with high TGFBR3 levels had an advanced Braak stage (Figure 6E). However, TGFBR3 expression showed weak association with age and there was also no statistical significance for gender and APOE genotype (Figures 6F–H).

### TGFBR3-Related Genomic Alterations

**FIGURE 7:**
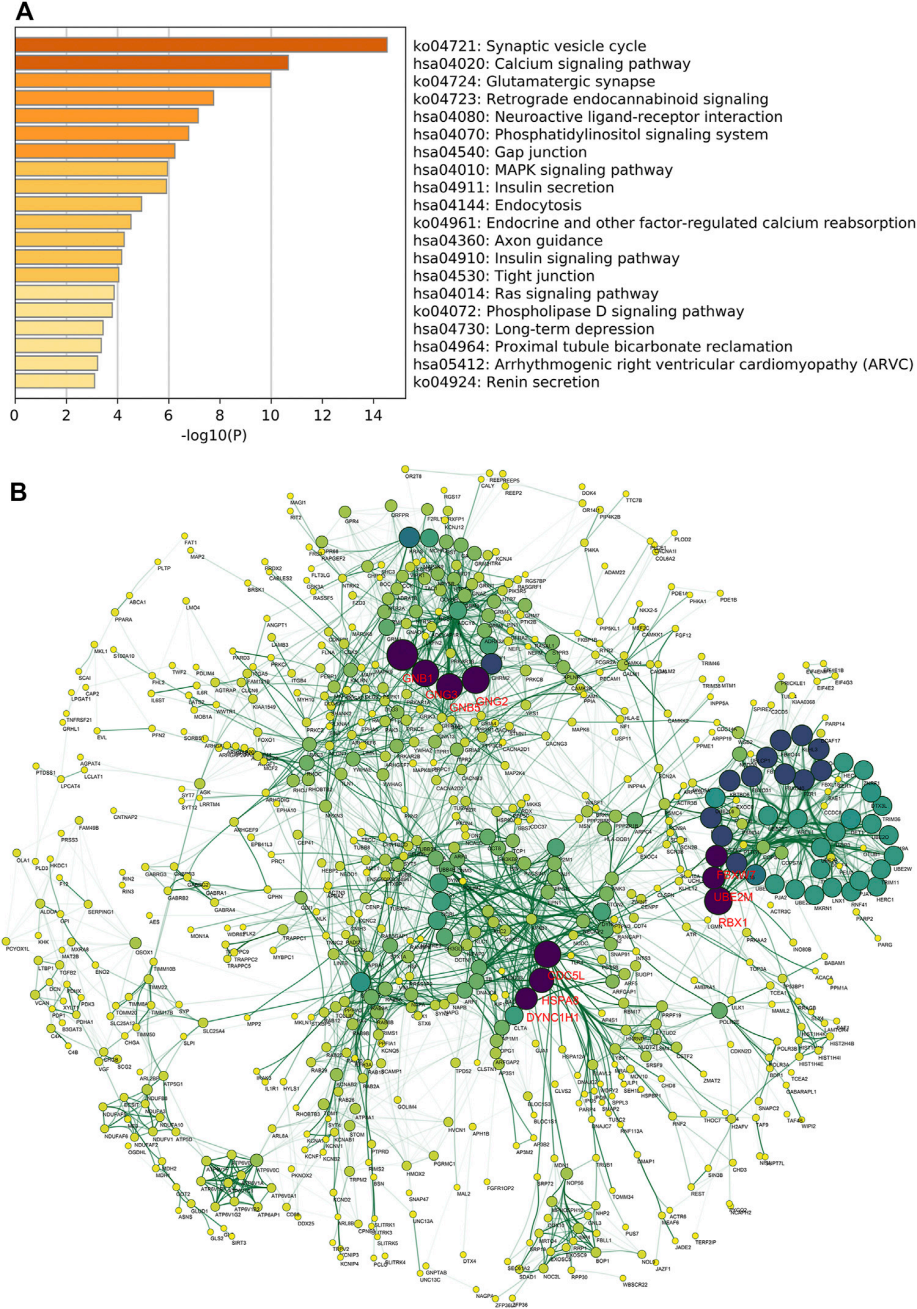
TGFBR3-related genomic alterations. A total of 1824 TGFBR3-related genes were identified based on the screening criteria |r|>0.6 and *p* < 0.05 from expression dataset 3. Then the KEGG pathway enrichment analysis was conducted by Metascape **(A)** Bar graph of the top 20 enriched KEGG pathways of TGFBR3-related genes, colored by *p*-values **(B)** The PPI network of TGFBR3-related genes was constructed by STRING v11.5 and visualized by Cytoscape v3.8.2. hub genes were identified from the network according to the degree value. Then the degree score of each gene in network was calculated by cytoHubba and the 10 genes with the highest degree score were identified as hub genes.

We next investigated the potential mechanism through which TGFBR3 exerted its functions in AD progression. Pearson correlation analysis was performed to obtain TGFBR3-correlated genes from expression dataset 3. According to the screening criteria |r|>0.6 and *p* < 0.05, 1824 genes were extracted and defined as TGFBR3-related genes. Pathway enrichment analysis revealed that these genes were closely related to several KEGG pathways, like Synaptic vesicle cycle, Calcium signaling pathway, Phosphatidylinositol signaling system, Phosphatidylinositol signaling system, MAPK signaling pathway and Insulin signaling pathway (Figure 7A). Subsequently, PPI network of the TGFBR3-related genes were constructed using STRING (Figure 7B). Based on the degree of each gene, we further identified 10 hub genes (GNB1, RBX1, GNG2, GNG3, CDC5L, GNB5, HSPA8, DYNC1H1, UBE2M and FBXW7) from the network (Supplementary Table S1). These 10 hub genes might be major regulators for TGFBR3 functions in AD.

### Validation of the Hub Genes

**FIGURE 8:**
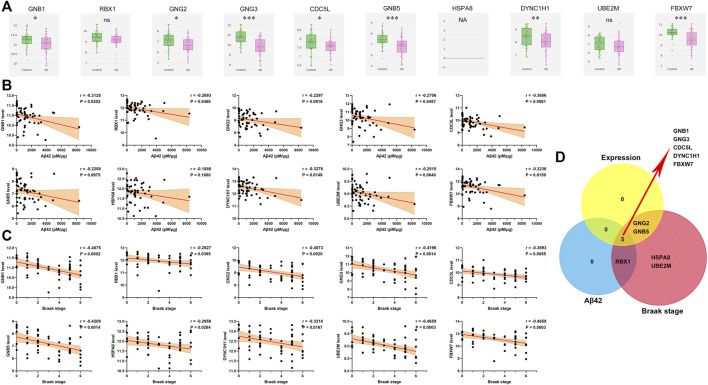
Validation of the hub genes **(A)** Expression levels of hub genes in AD and controls from AlzData database **(B)** Pearson correlation analysis was used to evaluate the association between and Aβ42 level and hub gene expression level in expression dataset 3 **(C)** Pearson correlation analysis was used to evaluate the association between and Braak stage and hub gene expression level in expression dataset 3 **(D)** Venn diagram analysis. NA, data are not available; ns *p* > 0.05, *p < 0.05, **p < 0.01, ***p < 0.001.

Utilizing gene expression data from AlzData, we observed that expression levels of GNB1, GNG2, GNG3, CDC5L, GNB5, DYNC1H1 and FBXW7 were higher in temporal cortex of AD patients than that of controls (Figure 8A). Patients with high levels of GNB1, RBX1, GNG3, CDC5L, DYNC1H1 and FBXW7 had an enhanced amount of Aβ42 level (Figure 8B). As shown in Figure 8C, a significant negative correlation of Braak stage was observed for all the 10 hub genes. Venn diagram analysis further verified GNB1, GNG3, CDC5L, DYNC1H1 and FBXW7 as potential downstream regulators of TGFBR3 (Figure 8D).

## Discussion

Numerous longitudinal studies have demonstrated that AD pathology develops decades preceding onset of clinical symptoms (Vermunt et al., 2019). Therefore, identification of candidate biomarkers will be helpful for early diagnosis and also provide potential therapeutic targets for AD treatment. With the fast development of microarray and high throughput sequencing technologies, more efforts are made to identify genomic biomarkers including AD. For example, Hokama et al. reported that diabetes mellitus-related genes were significantly altered in AD patients and AD mouse model that might be a result of AD pathology using expression dataset 1 (Hokama et al., 2014). Analysis of expression dataset 2 and 3 from AD and normal brain tissues identified many differentially expressed genes, like PCCB, ATF2, GFAP and CAMK4 (Liang et al., 2007; Marttinen et al., 2019). Gasparoni et al. discovered two novel methylation sites at the key AD risk genes of APP and ADAM17 based on methylation dataset 1 (Gasparoni et al., 2018). The above-mentioned studies offer the opportunity to figure out the AD molecular features and provide important resource for AD diagnosis and therapeutic intervention. Previous studies have shown that methylation has major roles in regulating gene expression. In this regard, methylation sites that could regulate gene expression levels are more likely to affect AD progression.

In the current study, we first performed an integrative analysis of multi-omics data in temporal cortex from AD patients. Based on both gene expression and DNA methylation profiling, we totally discovered 147 hypomethylated-upregulated genes and 262 hypermethylated-downregulated genes. Functional and pathway enrichment analysis revealed that methylation exert a broad influence on AD related processes, including chemical synaptic transmission, regulation of neurotransmitter receptor activity, and regulation of Aβ formation. Besides, chaperone-mediated autophagy (CMA), a lysosome-dependent selective degradation pathway, was also implicated in DNA methylation (Dice, 2007). CMA was reported to be suppressed at an early stage of AD and its activation could reduce the levels of Aβ plaques and tau phosphorylation, and ameliorate behavioral phenotype (Dou et al., 2020; Bourdenx et al., 2021; Xu X. et al., 2021; Caballero et al., 2021). These evidences support an unequivocal role for CMA in the development of AD. However, whether DNA methylation could regulate CMA is still unclear and more investigations are needed to study this possible association.

Applying CFG analysis and expression cross-validation in different brain regions, we identified six candidate risk genes for AD. NPTX2 belongs to the neuronal pentraxin family, whose promoter was frequently highly methylated in many solid tumors (Park et al., 2007; Shukla et al., 2013; Rasmussen et al., 2017; Alholle et al., 2013; Xu et al, 2021a). Decreased NPTX2 level has been reported to be associated with diverse neurological diseases, including Alzheimer’s disease, anxiety, vascular dementia, Parkinson’s disease and ischemia (Moran et al., 2008; Chang et al., 2018; Cai et al., 2019; Shao et al., 2020; Libiger et al., 2021). RTN1, the first identified member of the RTNs family, is predominantly expressed by neurons. Although RTN1 was found to be co-immunoprecipitated with BACE1, RTN1 deficiency showed no obvious effects on BACE1 activity (He et al., 2004; Shi et al., 2017). Sao et al. observed a reduced mRNA level of MEF2C in Japanese patients with AD, but its methylation rate had no significant difference between AD and control, different from our findings of MEF2C being hypermethylated-downregulated in AD (Sao et al., 2018). TGFBR3, also known as betaglycan, is the most abundantly expressed TGFBR. TGFBR3 could regulate TGF-β signaling pathway as either agonist or antagonist dependent on its the molecular form (Heldin and Moustakas, 2016; Vander et al., 2018). The transmembrane form is a TGF-β co-receptor and increases TGF-β signal transduction, while the soluble form serves as an antagonist for TGF-β ligands and inhibits TGF-β signaling. A recent study has shown a high expression of TGFBR3 in the hippocampus of AD patients, yet its biological function in AD has not been elucidated (Quan et al., 2020). Furthermore, we also observed UBE2N and IQGAP1 were aberrantly methylated and expressed in AD, but their potential roles are still unclear.

Aβ and phosphorylated tau accumulation are thought to be major neuropathogenic mediators of AD. Among the six candidate risk genes, we observed that only TGFBR3 expression was statistically associated with Aβ level in brain tissues. Its promoter-associated methylation site cg17074213 was identified as a potential biomarker of AD and was also strikingly associated with Braak stage. These observations suggest that hypermethylated TGFBR3 might be a potential regulator of Aβ generation. Expression dataset 3 further confirmed the association of TGFBR3 with Aβ level and Braak stage. Our data also showed that upregulated TGFBR3 might increase Aβ production through enhancing β- and γ-secretase activities.

Another major finding of this study is the identification of possible mechanisms underlying TGFBR3 function. Pathway enrichment analysis of TGFBR3-related genes demonstrated that TGFBR3 was strongly involved in Synaptic vesicle cycle, Calcium signaling pathway and Glutamatergic synapse, which have been well-defined in etiology of AD (Ovsepian et al., 2018; Alzheimer’s Association Calcium Hypothesis Workgroup, 2017; Conway, 2020). The MAPK family consists of several serine/threonine kinases that regulate diverse cellular responses, including Aβ-mediated toxicity (Abe and Saito, 2000; Ghasemi et al., 2015; Morroni et al., 2016; Iloun et al., 2020). Selective inhibition of certain MAPKs can ameliorate inflammatory response, synaptic dysfunction and cognitive decline (Maphis et al., 2016; Gee et al., 2020; Schnöder et al., 2020). Other pathways, like Insulin signaling pathway and Ras signaling pathway, have also been broadly implicated in AD development (Akhtar and Sah, 2020; Xiao et al., 2021). Collectively, these findings emphasize TGFBR3 as a widespread mediator of pathways related to AD progression.

From the PPI network, we further identified 5 hub genes (GNB1, GNG3, CDC5L, DYNC1H1 and FBXW7) as potential downstream regulators of TGFBR3. Among them, GNB1 and GNG3 belong to G protein submit family which acts as a molecular switch in the signal transduction of G protein coupled receptors. De novo pathogenic variants in GNB1 have been associated with many neurological diseases, such as developmental delay, dystonia, growth delay and seizures (Petrovski et al., 2016; Hemati et al., 2018). Mice with deficiency of GNG3 are lean and have seizures, and also show resistance to opioids and diet induced obesity (Schwindinger et al., 2004; Schwindinger et al., 2009). Mutations in DYNC1H1 gene could cause spinal muscular atrophy, intellectual disability, frontotemporal dementia and Parkinson’s disease (Willemsen et al., 2012; Szczałuba et al., 2018; Maretina et al., 2019; Mentis et al., 2021). Despite that FBXW7 has not been direct reported in AD, some potential evidences support the tent that FBXW7 might play a role in the pathogenesis of AD, including Aβ generation, neuronal apoptosis and cell senescence (Yang et al., 2021). These data collectively suggest that these hub genes might be involved in AD development. Future work will be need to elucidate their function during AD pathogenesis.

In summary, we conducted a comprehensive analysis utilizing multi-omics data, and identified some signature genes and cellular processes that may be involved in the AD pathogenesis. Our data also established an important role of the promoter hypomethylation of TGFBR3 in Aβ accumulation through enhancing β- and γ-secretase activities. Overall, these findings highlight TGFBR3 as a risk factor of AD patients and will help to develop diagnostic markers and therapeutic targets for AD treatment.

## Data Availability

The datasets presented in this study can be found in online repositories. The names of the repository/repositories and accession number(s) can be found in the article/Supplementary Material.
