# Supplementary material for: Promoter Hypomethylation of TGFBR3 as a Risk Factor of Alzheimer’s Disease: An Integrated Epigenomic-Transcriptomic Analysis
Source: Front Cell Dev Biol. 2022 Mar 2;9:825729. doi: 10.3389/fcell.2021.825729 (PMC8924075; doi:10.3389/fcell.2021.825729)
Supplement: Supplementary file 1 [file Table1.docx]

**Supplementary Table 1.** **Hub genes identified from the PPI network.**

| **Name** | **Degree** | **Betweenness** | **Closeness** |
| --- | --- | --- | --- |
| GNB1 | 69 | 26965.19 | 4.47E-04 |
| RBX1 | 60 | 23272.83 | 3.93E-04 |
| GNG2 | 59 | 13673.9 | 4.39E-04 |
| GNG3 | 58 | 12669.81 | 4.38E-04 |
| CDC5L | 58 | 111622.4 | 5.02E-04 |
| GNB5 | 56 | 12355.49 | 4.38E-04 |
| HSPA8 | 52 | 55489.99 | 4.81E-04 |
| DYNC1H1 | 45 | 37507.61 | 4.75E-04 |
| UBE2M | 45 | 15862.61 | 3.95E-04 |
| FBXW7 | 44 | 13989.38 | 3.94E-04 |
