# Supplementary figures and images for: Promoter Hypomethylation of TGFBR3 as a Risk Factor of Alzheimer’s Disease: An Integrated Epigenomic-Transcriptomic Analysis
Source: Front Cell Dev Biol. 2022 Mar 2;9:825729. doi: 10.3389/fcell.2021.825729 (PMC8924075; doi:10.3389/fcell.2021.825729)

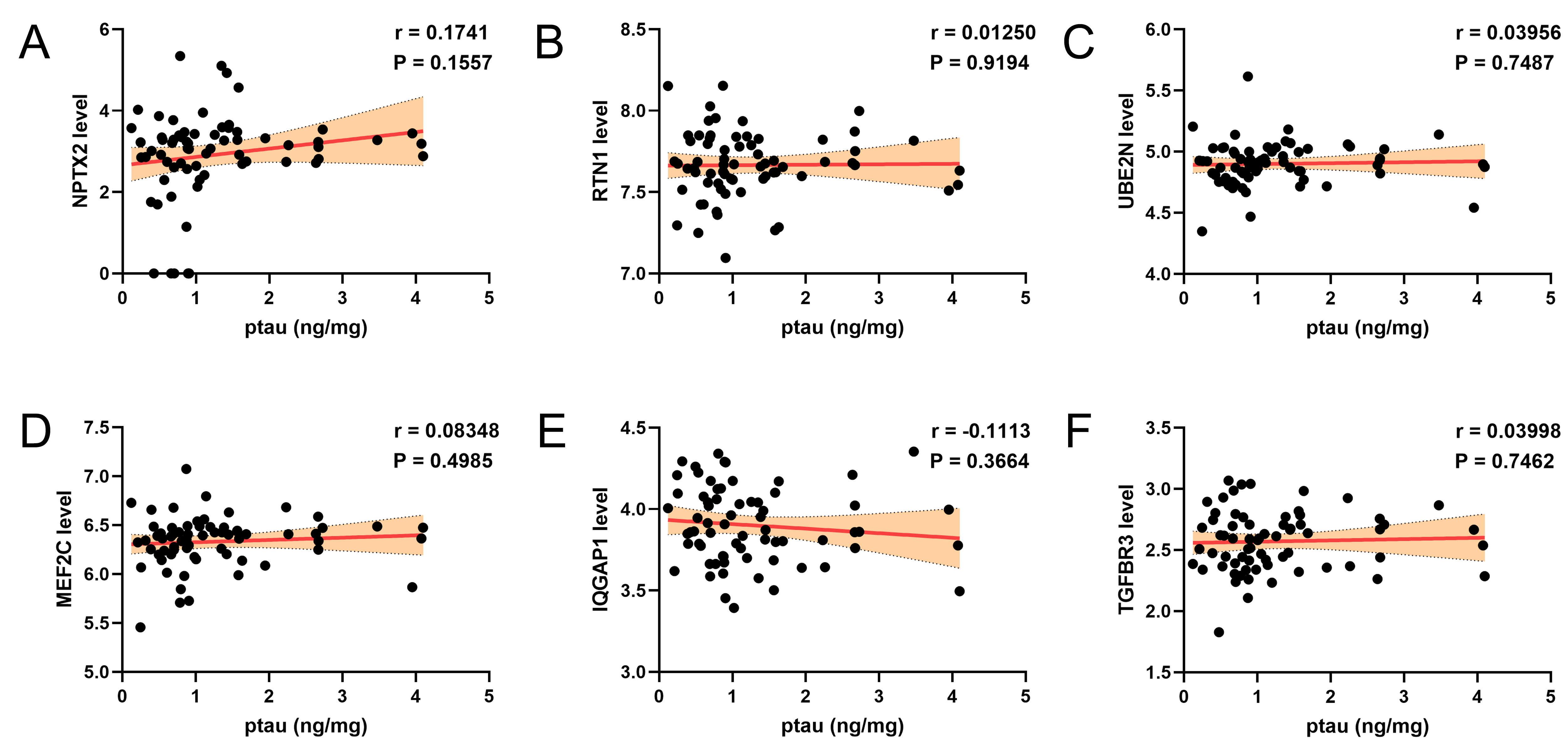

Supplement: Supplementary file 2 [file Image1.TIF]
